# Clinical indications for image-guided interventional procedures in the musculoskeletal system: a Delphi-based consensus paper from the European Society of Musculoskeletal Radiology (ESSR)—part VI, foot and ankle

**DOI:** 10.1007/s00330-021-08125-z

**Published:** 2021-08-25

**Authors:** Luca Maria Sconfienza, Miraude Adriaensen, Domenico Albano, Andrea Alcala-Galiano, Georgina Allen, Maria Pilar Aparisi Gómez, Giacomo Aringhieri, Alberto Bazzocchi, Ian Beggs, Vito Chianca, Angelo Corazza, Danoob Dalili, Miriam De Dea, Jose Luis del Cura, Francesco Di Pietto, Elena Drakonaki, Fernando Facal de Castro, Dimitrios Filippiadis, Salvatore Gitto, Andrew J Grainger, Simon Greenwood, Harun Gupta, Amanda Isaac, Slavcho Ivanoski, Monica Khanna, Andrea Klauser, Ramy Mansour, Silvia Martin, Vasco Mascarenhas, Giovanni Mauri, Catherine McCarthy, David McKean, Eugene McNally, Kalliopi Melaki, Carmelo Messina, Rebeca Mirón Mombiela, Ricardo Moutinho, Cyprian Olchowy, Davide Orlandi, Raquel Prada González, Mahesh Prakash, Magdalena Posadzy, Saulius Rutkauskas, Žiga Snoj, Alberto Stefano Tagliafico, Alexander Talaska, Xavier Tomas, Violeta Vasilevska Nikodinovska, Jelena Vucetic, David Wilson, Federico Zaottini, Marcello Zappia, Marina Obradov

**Affiliations:** 1 Unit of Diagnostic and Interventional Radiology, IRCCS Istituto Ortopedico Galeazzi, Via Riccardo Galeazzi 4, 20161 Milan, Italy; 2 Dipartimento di Scienze Biomediche per la Salute, Università degli Studi di Milano, Milan, Italy; 3 Department of Medical Imaging, Zuyderland Medical Center, Sittard-Geleen, Heerlen, Brunssum, Kerkrade, the Netherlands; 4 Sezione di Scienze Radiologiche, Dipartimento di Biomedicina, Neuroscienze e Diagnostica Avanzata, Università degli Studi di Palermo, Palermo, Italy; 5 Hospital Universitario 12 de Octubre, Madrid, Spain; 6 St Luke’s Radiology Oxford Ltd, Oxford, UK; 7 University of Oxford, Oxford, UK; 8 Department of Radiology, Auckland City Hospital, Auckland, New Zealand; 9 Department of Radiology, Hospital Vithas Nueve de Octubre, Valencia, Spain; 10 Diagnostic and Interventional Radiology, Department of Translational Research and New Technologies in Medicine and Surgery, University of Pisa, Pisa, Italy; 11 Diagnostic and Interventional Radiology, IRCCS Istituto Ortopedico Rizzoli, Bologna, Italy; 12 Analytic Imaging, Edinburgh, UK; 13 Ospedale Evangelico Betania, Napoli, Italy; 14 Clinica di Radiologia EOC IIMSI, Lugano, Switzerland; 15 School of Biomedical Engineering & Imaging Sciences, King’s College London, London, UK; 16 Studio MSK, Belluno, Italy; 17 Hospital Universitario Donostia, San Sebastian, Spain; 18 Dipartimento di Diagnostica per Immagini, Pineta Grande Hospital, Castel Volturno, Italy; 19 MSK Radiology Services, Heraklion, Crete, Greece; 20 Hospital General Universitario de Valencia, Valencia, Spain; 21 2nd Department of Radiology, University General Hospital “ATTIKON” Medical School, National and Kapodistrian University of Athens, Haidari, Athens, Greece; 22 Dipartimento di Scienze Biomediche per la Salute, Università degli Studi di Milano, Milan, Italy; 23 Cambridge University Hospitals, Cambridge, UK; 24 Mid Cheshire Hospitals NHS Foundation Trust, Cheshire, UK; 25 Leeds Teaching Hospitals, Leeds, UK; 26 Guy’s and St Thomas’ Hospitals, London, UK; 27 Department of Radiology, Special Hospital for Orthopedic Surgery and Traumatology, St. Erazmo –, Ohrid, North Macedonia; 28 Ss. Cyril and Methodius University of Skopje, Skopje, North Macedonia; 29 Imperial College Healthcare NHS Trust, London, UK; 30 Department of Radiology, Medical University Innsbruck, Innsbruck, Austria; 31 Oxford Musculoskeletal Radiology, Oxford University Hospitals, Oxford, UK; 32 Hospital Universitario Son Llatzer, Palma, Spain; 33 Hospital da Luz, Musculoskeletal Imaging Unit, Lisbon, Portugal; 34 AIRC, Advanced Imaging Research Consortium, Lisbon, Portugal; 35 Division of Interventional Radiology, Istituto Europeo di Oncologia, Milan, Italy; 36 Department of Oncology and Hemato-Oncology, University of Milano, Milan, Italy; 37 Oxford Musculoskeletal Radiology, Oxford, UK; 38 Buckinghamshire Healthcare NHS Trust, Aylesbury, UK; 39 Department of Radiology, Charité-Universitätsmedizin Berlin, Berlin, Germany; 40 Department of Radiology, Herlev Gentofte Hospital, Herlev, Denmark; 41 Hospital de Loulé, Loulé, Portugal; 42 Department of Oral Surgery, Wroclaw Medical University, Wroclaw, Poland; 43 Department of Radiology, Ospedale Evangelico Internazionale, Genoa, Italy; 44 Ribera Povisa Hospital, Vigo, Spain; 45 Post Graduate Institute of Medical Education & Research (PGIMER), Chandigarh, India; 46 Indywidualna Praktyka Lekarska Magdalena Posadzy, Poznan, Poland; 47 Department of Radiology, Lithuanian University of Health Sciences, Kaunas, Lithuania; 48 Institute of Radiology, University Medical Centre Ljubljana, Zaloska 7, 1000 Ljubljana, Slovenia; 49 Faculty of Medicine, University of Ljubljana, Ljubljana, Slovenia; 50 Department of Health Sciences, University of Genova, Genoa, Italy; 51 IRCCS Ospedale Policlinico San Martino, Genova, Italy; 52 Department of Radiology, AUVA Trauma Center Vienna, Vienna, Austria; 53 Radiology Dpt. MSK Unit. Hospital Clinic (CDIC), University of Barcelona (UB), Barcelona, Spain; 54 University Institute of Radiology, Skopje, Macedonia; 55 Ss. Cyril and Methodius University in Skopje, Skopje, Macedonia; 56 Radiology Department, Hospital ICOT Ciudad de Telde, Las Palmas, Spain; 57 Department of Medicine and Health Sciences, University of Molise, Campobasso, Italy; 58 Varelli Institute, Naples, Italy; 59 Department of Radiology, Sint Maartenskliniek, Nijmegen, The Netherlands

**Keywords:** Interventional radiology, Ultrasonograph, Ankle, Foot, Achilles tendon

## Abstract

**Objectives:**

Clarity regarding accuracy and effectiveness for interventional procedures around the foot and ankle is lacking. Consequently, a board of 53 members of the Ultrasound and Interventional Subcommittees of the European Society of Musculoskeletal Radiology (ESSR) reviewed the published literature to evaluate the evidence on image-guided musculoskeletal interventional procedures around this anatomical region.

**Methods:**

We report the results of a Delphi-based consensus of 53 experts from the European Society of Musculoskeletal Radiology who reviewed the published literature for evidence on image-guided interventional procedures offered around foot and ankle in order to derive their clinical indications. Experts drafted a list of statements and graded them according to the Oxford Centre for evidence-based medicine levels of evidence. Consensus was considered strong when > 95% of experts agreed with the statement or broad when > 80% but < 95% agreed. The results of the Delphi-based consensus were used to write the paper that was shared with all panel members for final approval.

**Results:**

A list of 16 evidence-based statements on clinical indications for image-guided musculoskeletal interventional procedures in the foot and ankle were drafted after a literature review. The highest level of evidence was reported for four statements, all receiving 100% agreement.

**Conclusion:**

According to this consensus, image-guided interventions should not be considered a first-level approach for treating Achilles tendinopathy, while ultrasonography guidance is strongly recommended to improve the efficacy of interventional procedures for plantar fasciitis and Morton’s neuroma, particularly using platelet-rich plasma and corticosteroids, respectively.

**Key Points:**

• *The expert panel of the ESSR listed 16 evidence-based statements on clinical indications of image-guided musculoskeletal interventional procedures in the foot and ankle.*

*• Strong consensus was obtained for all statements.*

*• The highest level of evidence was reached by four statements concerning the effectiveness of US-guided injections of corticosteroid for Morton’s neuroma and PRP for plantar fasciitis.*

**Supplementary Information:**

The online version contains supplementary material available at 10.1007/s00330-021-08125-z.

## Introduction

Musculoskeletal interventional procedures are routinely performed around the foot and ankle, including injections and variable interventions on tendons, bursae, plantar fascia, nerves, and joints [1–3]. Some of them are superficial and can be easily approached using palpation, but others generally require imaging, specifically ultrasonography (US), to guide the procedure and to accurately deliver the medications [4–6]. Indeed, foot and ankle musculoskeletal structures can be very small and located close to neurovascular bundles that can be damaged during the procedures [7–9]. No clear guidelines have been produced concerning the use of imaging guidance for musculoskeletal interventional procedures around the foot and ankle. Clarity is needed regarding the accuracy and effectiveness of these interventions. These procedures are widely adopted by different physicians, but there is no consensus on which image-guided procedures should be considered first, particularly when involving relatively novel approaches, including hyaluronic acid (HA), regenerative medications like platelet-rich plasma (PRP), or ablation (e.g., radiofrequency and cryoablation of Morton’s neuroma) [10–12]. The Ultrasound and the Interventional Subcommittees of the European Society of Musculoskeletal Radiology (ESSR) carried out a collaborative task, with the support of its Research Committee, to analyze the published literature on image-guided musculoskeletal interventional procedures in the lower limb to establish clinical indications (upcoming publication in *Eur Radiol*). We report the evidence-based statements for clinical indications of image-guided musculoskeletal interventional procedures around the foot and ankle listed by an expert board of the ESSR after a Delphi method performed a review of current literature.

## Materials and methods

Institutional Review Board approval was not needed as no patients were directly involved. This paper arises from the review of published evidence on image-guided interventional musculoskeletal procedures around the foot and ankle. Similar to previous ESSR consensus papers [13–16], a literature-based Delphi method of review was used. It includes multiple rounds of evaluation of the existing literature to assess the opinion of a panel of experts on this topic. A list of statements were discussed and drafted to reach a final shared agreement [17]. We used the AGREE II tool to guarantee the quality of the working flow [18]. The different steps of the Delphi method are thoroughly explained and reported as supplementary material.

## Results

1. **Image-guided injections are safe and feasible to treat Achilles degenerative tendinopathy (AT), but there is insufficient evidence from randomized controlled trials (RCT) to support them over conservative therapies.**

Level of evidence: 1

Agree, *n = *53; disagree, *n *= 0; abstain, *n = *0. Agreement = 100%

Both the Cochrane Review of 2015 [19] and Maffulli’s systematic review [20] concluded that there is insufficient evidence from RCTs to support the use of injection therapies. The evidence has not changed since then. Previous meta-analyses of RCTs demonstrated no superiority of US-guided PRP injections over placebo [11, 21–25]. Notably, PRP injections and dry needling have similar short-term results with weekly injections of PRP or dry needling for 3 weeks having shown no clinical difference at 3 or 6 months [26]. A systematic review reported local US-guided corticosteroid injections have no therapeutic role to treat AT [27] and subcutaneous fat atrophy and tendon damage may occur [28]. A systematic review of many high-quality RCTs showed corticosteroid injections reduced pain in the short term compared with other interventions, but this effect was reversed in the intermediate and long term [29]. There is controversial evidence that bone marrow aspirate and stem cells help in the treatment of AT, with few studies suggesting a potential short-term clinical improvement, with no role for imaging to predict or assess the response [30, 31]. A meta-analysis reported that prolotherapy may be safe and effective for AT [32]. However, long-term studies and RCTs are still missing and one RCT found no clinical difference between prolotherapy and the control group. No significant pain variation was found between polidocanol and lidocaine injection for AT [33].

High-volume injections seem to be effective at treating AT, but confounding factors such as corticosteroid, aprotinin, dry needling, or eccentric exercise prevent assessment of this method alone [34, 35].

2. **Image-guided anesthetic-corticosteroid injections into the anterior and posterior tibial tendon sheaths are safe and may provide effective diagnosis and treatment in patients with anterior and posterior tibial tendon tenosynovitis.**

Level of evidence: 3

Agree, *n* = 53; disagree, *n* = 0; abstain, *n* = 0. Agreement = 100%

Several studies reported complete or near-complete prolonged symptom relief after anesthetic-corticosteroid injections for tenosynovitis unresponsive to conservative management [36–40]. A study [39] using US guidance successfully demonstrated excellent improvement of the tendon sheath effusion and synovial hypertrophy around the anterior and posterior tibial tendon 4 weeks after injection.

3. **US-guided corticosteroid injections are more effective than palpation-guided injections to treat plantar fasciitis (PF) providing significant short-term pain relief, particularly when combined with strength training and stretching.**

Level of evidence: 2

Agree, *n* = 53; disagree, *n* = 0; abstain, *n* = 0. Agreement = 100%

A meta-analysis included 5 RCTs with 149 patients and concluded that US-guided injections were superior with regard to VAS, tenderness threshold, response rate, plantar fascia thickness, and hypoechogenicity. There was no difference in heel pad thickness and heel tenderness index [41]. A comparative trial concluded that device-assisted US-guided injection for treating PF was superior to palpation-guided injection [42]. US-guided corticosteroid injections are associated with lower heel pain recurrence when compared to palpation guidance. Comparative trials of US-guided corticosteroid injections versus placebo reported greater pain relief in the corticosteroid arm lasting up to 12 weeks [43]. Case series in the literature report efficacy of corticosteroid injections for PF with objective measurement of the therapeutic effect on proximal PF without significant deterioration of heel pad mechanical properties [44–47]. Combining corticosteroid injections with strength training and stretching is superior both in the short- and in the long term and can be recommended as a first-line treatment in patients with PF [48].

4. **US-guided PRP injections for PF are safe and provide significant pain relief in chronic PF, with better clinical outcome at mid- and long-term follow-up if compared with corticosteroid injections.**

Level of evidence: 1

Agree, *n* = 53; disagree, *n* = 0; abstain, *n* = 0. Agreement = 100%

Four meta-analysis studies having compared US-guided PRP and corticosteroid support the use of PRP instead of corticosteroids, highlighting the favorable and long-lasting clinical outcomes in patients with chronic PF [10, 49–51].

5. **The effectiveness of US-guided injections with ozone, hyaluronic acid, or botulinum toxin type A has not still sufficiently proven to be recommended for PF.**

Level of evidence: 3

Agree, *n* = 53; disagree, *n* = 0; abstain, *n* = 0. Agreement = 100%

Limited number of outcome-based investigation studies reported the effectiveness of those methods to treat painful PF without inducing fat pad atrophy [52–54]. Most studies are case series or trials comparing different doses of the same substance [54], but no comparative studies are available.

6. **US and fluoroscopy guidance improves the accuracy of joint injections in the foot and ankle although these procedures can be safely performed with palpation alone.**

Level of evidence: 4

Agree, *n* = 53; disagree, *n* = 0; abstain, *n* = 0. Agreement = 100%

Intra-articular injections of the foot and ankle using palpation and US were conducted on a cadaver model and were both 100% accurate [55]. The risk of extravasation into the ankle or peroneal tendon sheath is higher when injections are unguided [56]. When injections are used for diagnostic purposes and surgical decision-making, especially in abnormal joints, imaging guidance is useful. The use of US or fluoroscopy significantly improved the accuracy of injections into the tarsometatarsal joints [55]. A cadaveric study on talonavicular joint injection accuracy showed that the needle was correctly positioned in all US-guided injections, while it was misplaced in all cases of blind injections [57]. In juvenile idiopathic arthritis patients with ankle synovitis, image-guided intra-articular corticosteroid injections resulted in longer remission time when compared with palpation guidance [58].

7. **Image-guided corticosteroid injections for midfoot joint osteoarthritis might provide short-term improvement, but further studies are warranted to support their clinical use.**

Level of evidence: 4

Agree, *n* = 53; disagree, *n* = 0; abstain, *n* = 0. Agreement = 100%

Two uncontrolled studies of intra-articular corticosteroid injections for midfoot osteoarthritis led to short-term improvement with poor long-term outcome [59, 60]. Symptom improvement reported at 3–4 months was generally not sustained at 12 months [59, 60]. Larger prospective RCTs are needed.

8. **PRP and prolotherapy are safe methods to treat osteochondral lesions of the talus with promising results, but evidence on efficacy is limited.**

Level of evidence 4

Agree, *n = *53; disagree, *n* = 0; abstain, *n* = 0. Agreement = 100%

PRP and prolotherapy are shown to be safe methods to treat talus osteochondral lesions and might be considered treatment options for younger patients and for patients with early stage and small lesions, as reported in a single retrospective cohort study including 49 patients (*n* = 27 prolotherapy, *n* = 22 PRP) [61]. Prolotherapy is cheaper and less invasive [61]. However, further studies are still required.

9. **Intraarticular foot and ankle anesthetic injections performed under imaging guidance offers precise information about pain source.**

Level of evidence: 4

Agree, *n* = 53; disagree, *n* = 0; abstain, *n* = 0. Agreement = 100%

Fluoroscopically guided foot and ankle joint anesthetic injections aid diagnosis and have a potential for targeted therapy when combined with corticosteroids. Large and small joints in the foot and ankle are readily accessed [62, 63]. Anesthetic response for surgical planning is controversial [64, 65], as many foot joints are interconnected, with a potential confounding effect, but when correlated with magnetic resonance or computed tomography arthrography, they may help confirm the diagnosis [63, 66].

10. **US-guided tarsal tunnel decompression is a feasible procedure in cadavers, but clinical value is unknown.**

Level of evidence: 4

Agree, *n* = 52; disagree, *n* = 1; abstain, *n* = 0. Agreement = 98%

US-guided tarsal tunnel decompression associated with flexor retinaculum release was feasible in a cadaveric study without any vascular or neurological damage [67]. However, no data is available about in-vivo feasibility and efficacy.

11. **US-guided injections of bursae around the foot are technically feasible, but clinical value is unknown.**

Level of evidence: 5

Agree, *n* = 53; disagree, *n* = 0; abstain, *n* = 0. Agreement = 100%

Foot bursae aspiration and injection could be performed in a wide series of rheumatologic and degenerative conditions [68], but evidence about the interventional approach for foot bursitis is poor. There is minor evidence for intermetatarsal bursa US-guided corticosteroid injection feasibility and its therapeutic role in Morton’s syndrome [69].

12. **US-guided deep retrocalcaneal bursa injections are feasible.**

Level of evidence: 4

Agree, *n* = 53; disagree, *n = *0; abstain, *n* = 0. Agreement = 100%

Deep retrocalcaneal bursa injections are feasible and effective both under US [70] and fluoroscopy [71] guidance but there is more evidence for US guidance [72]. A direct comparison between the two methods is not available, and the evidence about injection indications is weak. Local US-guided corticosteroid injection of the retrocalcaneal bursa improves bursitis symptoms but risks Achilles tendon rupture [73].

13. **US-guided corticosteroid injections are the most effective image-guided interventional procedure to improve pain in patients with Morton’s neuroma, especially in the first 3 months.**

Level of evidence: 1

Agree, *n = *53; disagree, *n* = 0; abstain, *n* = 0. Agreement = 100%

A systematic review identified eight different types of interventions for Morton’s neuroma. The most frequent procedure was US-guided corticosteroid (7 studies) and sclerosing injections (7 studies). A meta-analysis of two RCTs conducted in the same study found that US-guided corticosteroid injections decreased pain more than controls. Other RCTs reported the efficacy of mobilization or extracorporeal shockwave therapy versus control. Treatment success was evaluated for extracorporeal shockwave therapy versus control and for corticosteroids versus footwear padding. Sclerosing and Botox injections, radiofrequency ablation, and cryoneurolysis had been investigated only by case studies with limited methodological quality [74]. Results from a patient-blinded trial [75] comparing US-guided injection of either corticosteroid-anesthetic or anesthetic alone demonstrated that the corticosteroid group had significantly better clinical improvement at 1 and 3 months. Controversial results have been reported by a double-blinded RCT [76] comparing three US-guided injections of either corticosteroid-anesthetic or local anesthetic alone, showing no clinical differences between the groups at 3 and 6 months.

14. **US guidance improves the effectiveness of different interventional procedures for Morton’s neuroma if compared to palpation guidance, particularly for corticosteroid injection.**

Level of evidence: 1

Agree, *n* = 53; disagree, *n* = 0; abstain, *n* = 0. Agreement = 100%

When compared to palpation guidance, US was proven to increase the effectiveness of interventional procedures in patients with Morton’s neuroma. A systematic review [77] reported that US guidance may produce better short- and long-term pain relief after corticosteroid injection and can reduce surgical referral rate, and need for additional procedures after ethanol injection. An RCT [78] concluded that US-guided injection of Morton’s neuroma provides significant improvement at 45, 60, and 90 days compared with palpation-guided injections. Multiple studies reported the use of US guidance using different types of intervention with satisfactory results: corticosteroids [69, 75, 76, 79–81], alcohol [82–88], radiofrequency [89–91], and hyaluronic acid [92]. A case study concluded that the injection of Morton’s neuroma was better tolerated via a dorsal approach and the preliminary use of local anesthetic did not confer any benefit [93]. There is conflicting evidence about the relationship between Morton’s neuroma size and efficacy of US-guided anesthetic and/or corticosteroid injection. A case-control study [94] concludes that size and age appear to be predictors for further treatment within 2 years from corticosteroid injection. Another case study [95] reported that the effectiveness of corticosteroid injection appears to be more significant and long-lasting for lesions < 5 mm. However, results from a patient-blinded RCT [75] comparing corticosteroid-anesthetic versus anesthetic alone reported that the neuroma size did not significantly influence the treatment effect.

15. **US-guided ethanol injection of Morton’s neuroma is relatively safe and well-tolerated, but further investigations are required to clearly demonstrate its clinical value prior to supporting this procedure.**

Level of evidence: 2

Agree, *n* = 53; disagree, *n* = 0; abstain, *n* = 0. Agreement = 100%

A systematic review [88] found that ethanol injections appear to be safe, although some papers report short-term side effects due to an inflammatory reaction. Ethanol concentration and US guidance versus unguided injections vary. Evidence suggests ethanol has a sclerosing histological effect on the interdigital nerve but all reviewed studies had methodological flaws causing bias and poor evidence. A level 3 [87] and multiple level 4 studies [82, 83, 85, 86, 88] explored treatment with US-guided ethanol injection, with good tolerance and high success rate. A prospective case series [84] demonstrated that short-term benefits from ethanol injection exist with considerable morbidity and no long-term benefit. Pain and satisfaction scores showed significant deterioration after 5 years. Fluoroscopic and electroneurographic guidance gave a success rate (defined as free of pain in daily life) of more than 82% per single ethanol injection with no recurrence over 5 years [96].

16. **Image-guided thermal ablation of Morton’s neuroma is safe with promising initial results and might reduce the need for surgery in the short term.**

Level of evidence: 4

Agree, *n* = 53; disagree, *n* = 0; abstain, *n* = 0. Agreement = 100%

Case series reported the use of radiofrequency ablation as a safe and efficient potential treatment for Morton’s neuroma [90, 91]. A case series [89] reported successful symptom relief in > 85% of cases, and only 10% of patients needed surgery in the short term. Radiofrequency ablation has the disadvantage of being more expensive compared to corticosteroid injections. Neurolysis is a treatment used for chronic peripheral pain, with cryotherapy being recognized as a well-tolerated procedure among the different methods of nerve ablation. MR-guided Morton’s neuroma cryoablation has been reported by Cazzato et al as a feasible and tolerated technique with initially promising results requiring further investigation [12]. Friedman and colleagues reported good clinical results from a retrospective study on 20 patients subjected to US-guided cryoablation of Morton’s neuroma [97].

## Discussion

This panel listed 16 evidence-based statements on clinical indications of image-guided musculoskeletal interventional procedures in the foot and ankle, with strong consensus reached for all statements. The highest level of evidence was reached by four statements concerning the effectiveness of US-guided injections of corticosteroid for Morton’s neuroma and PRP for PF, and also concerning the limited role of injection therapies in AT.

Despite the safety and efficacy of image-guided injections for AT, the superiority of these procedures over conservative therapies has not been proven, leading us to consider them a second-level approach for treating patients with AT (statement #1).

Regarding PF, prospective RCTs demonstrated the added value of US guidance to improve the clinical outcome of interventional procedures over blinded injections (statement #3). The highest level of evidence paper showed that US-guided PRP injections have better and long-lasting effects than US-guided corticosteroid injections in patients with PF (statement #4). In patients with Morton’s neuroma, the highest level of evidence papers showed that US guidance improves the efficacy of several interventional procedures (statement #14), particularly corticosteroid injections used to improve pain in the short-/mid-term (statement #13). Although ethanol injections, radiofrequency, and cryoablation seem to be safe alternatives for treating Morton’s neuroma, the clinical value of these interventions still needs further clarification (statements #15 and #16).

Anecdotal reports and small case series have shown the feasibility, safety, and effectiveness of image-guided injections of ankle/foot joints (statements #6–9) and bursae (statements #11–12), with too limited evidence to support these in routine clinical practice.

In conclusion, 16 statements about image-guided musculoskeletal interventional procedures around the foot and ankle have been produced by an expert panel of the ESSR. Image-guided interventions should not be considered a first-level approach for AT. US is strongly recommended as a guidance for interventions for PF and Morton’s neuroma to improve the effectiveness of the procedures, particularly using PRP and corticosteroids, respectively.

## Supplementary information

ESM 1 (DOCX 20 kb)

## Abbreviations

AT: Achilles degenerative tendinopathy
ESSR: European Society of Musculoskeletal Radiology
HA: Hyaluronic acid
PF: Plantar fasciitis
PRP: Platelet-rich plasma
RCT: Randomized controlled trials
US: Ultrasonography

## Declarations

### Guarantor

The scientific guarantor of this publication is Luca Maria Sconfienza, MD, PhD.

### Statistics and biometry

No complex statistical methods were necessary for this paper.

### Methodology

• Multicentre study
