# Supplementary material for: Clinical indications for image-guided interventional procedures in the musculoskeletal system: a Delphi-based consensus paper from the European Society of Musculoskeletal Radiology (ESSR)—part VI, foot and ankle
Source: Eur Radiol. 2021 Aug 25;32(2):1384–94. doi: 10.1007/s00330-021-08125-z (PMC8794903; doi:10.1007/s00330-021-08125-z)
Supplement: Supplementary file 1 — (DOCX 20 kb) [file 330_2021_8125_MOESM1_ESM.docx]

**Supplementary material to “Clinical indications for image guided interventional procedures in the musculoskeletal system: a Delphi-based consensus paper from the European Society of Musculoskeletal Radiology (ESSR) — part VI, foot and ankle”**

The Delphi based method review of evidence is divided in the following parts:

1. *Expert selection*

A board of 53 radiologists from 16 Countries (Austria, Belgium, Denmark, Germany, Greece, India, Italy, Lithuania, New Zealand, North Macedonia, Poland, Portugal, Slovenia, Spain, The Netherlands, United Kingdom), with experience in musculoskeletal interventional procedures and in the scientific assessment of medical articles, were identified among the members of the Ultrasound and Interventional Subcommittees of the ESSR and divided in small groups to assess different topics, specifically image-guided interventions on tendons, fascia, bursae, and joints of the foot and ankle.

1. *Literature search, statement drafting, and level of evidence*

All reviewers used the search terms relevant to the specific topic assigned to their group to search published studies up to December 2020 on the major online databases (MEDLINE, Web of Science, EMBASE, and Google). They included also any other articles that deserved to be included in this review, also screening the references of retrieved papers for any additional studies. Then, all groups prepared a draft of statements concerning their assigned procedures using the criteria of the Oxford Center of Evidence-Based Medicine in 2011 to identify the correct level of evidence for each drafted statement [1].

1. *Questionnaire preparation and consensus process*

The coordinator of this task revised the statements and shared a tool (Google Forms, Google LLC) sending out an online link via email to all experts selected in this project. All reviewers were invited to agree, disagree, or abstain for all drafted statements. They could also provide any comments concerning level of evidence, content and form of each statement. All replies and any comments were retrieved and saved in an electronic spreadsheet (Microsoft Excel, Microsoft). Then, the coordinator analysed all answers of the first round of review, modified the statements accordingly, and a second round with the same modalities was set up. After the second round, persisting conflicts regarding the statements were solved or kept via emails specifically sent to the involved experts.

1. *Data analysis and paper drafting*

After both rounds of discussion, the final statements were shared to reach the consensus opinion. The consensus was regarded to be strong when more than 95% of reviewers agreed or broad when more than 80% but less than 95% agreed on the statement [2]. The results of this consensus were used to build up the article and was shared with all panel members for final approval.

**References to supplementary material**

1. Oxford Center of Evidence Based Medicine. OCEBM Levels of Evidence 2016 v. 2.1. 2016 <https://www.cebm.net/wp-content/uploads/2014/06/CEBM-Levels-of-Evidence-2.1.pdf> Accessed on 21 February 2021
2. Săftoiu A, Gilja OH, Sidhu PS, et al (2019) The EFSUMB Guidelines and Recommendations for the Clinical Practice of Elastography in Non-Hepatic Applications: Update 2018. Ultraschall Med. doi: 10.1055/a-0838-993
